# A novel mechanism of lncRNA and miRNA interaction: CCAT2 regulates miR-145 expression by suppressing its maturation process in colon cancer cells

**DOI:** 10.1186/s12943-017-0725-5

**Published:** 2017-09-30

**Authors:** Yingjie Yu, Pratima Nangia-Makker, Lulu Farhana, Adhip P. N. Majumdar

**Affiliations:** 1 Department of Veterans Affairs Medical Center, 4646 John R, Detroit, MI 48201 USA; 2 Karmanos Cancer Institute, Detroit, MI 48201 USA; 3 Departments of Internal Medicine, Wayne State University, Detroit, MI 48201 USA

**Keywords:** miRNA biogenesis, lncRNA-miRNA crosstalk, cancer stem cells

## Abstract

**Background:**

Although both long and micro RNAs are emerging as important functional components in colorectal cancer (CRC) progression and metastasis, the mechanism of their interaction remains poorly understood. CCAT2 (Colon cancer-associated transcript-2), a long noncoding RNA (lncRNA), has been reported to be over-expressed in CRC and is found to promote tumor growth. miRNAs, a class of naturally occurring short RNAs negatively control the expression of target genes by cleaving mRNA or through translation repression. Recently, we reported that miR-145 and miR-21 cooperate to regulate colon cancer stem cell (CSC) proliferation and differentiation. Considering that CCAT2 is mainly located in the nucleus and miRNA maturation process begins in the nucleus, we *hypothesize* that CCAT2 selectively blocks miR-145 maturation process, resulting in decreased mature miR-145 affecting colon CSC proliferation and differentiation.

**Methods:**

The levels of CCAT2 were manipulated by transfection of CCAT2 expression plasmid or knockdown by siRNA or by CRISPR/Cas9. Quantitative RT-PCR was performed to examine the expression of CCAT2 and pri-, pre- and mature miR-145/21. Fluorescence in situ hybridization (FISH) was used to visualize *CCAT2* in the cells. In vitro processing of pri-miRNA-145 was performed using T7 RNA polymerase and recombinant human Dicer.

**Results:**

We have observed that modulated expression of CCAT2 regulates the expression of miR-145 in colon cancer HCT-116 and HT-29 cells. Knockout of CCAT2 increases miR-145 and negatively regulates miR-21 in HCT-116 cells, impairs proliferation and differentiation. In contrast, stable up-regulation of CCAT2 decreases mature miR-145 and increases the expression of several CSC markers in colon cancer cells. We have also observed that CCAT2 is enriched in the nucleus and correlates with the expression of pre-miR-145 but not pre-miR-21 in HCT-116 cells. These results indicate CCAT2 selectively blocks miR-145 maturation by inhibiting pre-miR-145 export to cytoplasm. Further, we revealed that CCAT2 blocks cleavage of pre-miR-145 by Dicer in vitro.

**Conclusions:**

Our results identify CCAT2 as a negative regulator of miRNA-145 biogenesis, and expose a novel mechanism of lncRNA-miRNA crosstalk.

**Electronic supplementary material:**

The online version of this article (10.1186/s12943-017-0725-5) contains supplementary material, which is available to authorized users.

## Background

Recent studies indicate that a vast majority of transcribed non-coding RNAs (ncRNAs) play a key role in regulating development and growth of tumor. The ncRNA can be divided into two groups, small and long noncoding RNAs. The RNAs which are less than 200 nucleotides in length are considered small RNA [1]. Current small RNAs correspond to approximate 18–29 nucleotides in length and common members include small interfering RNAs (siRNAs), microRNA (miRNAs) and PIWI associated RNAs (piRNAs) [2]. Long noncoding RNAs (lncRNAs) are more than 200 nucleotides long diverse class of transcribed RNA molecules that do not encode proteins but regulate expression of coding genes.

miRNAs comprise a broad class of small (19–22 nucleotide) endogenous small ncRNAs that negatively control the expression of target genes by cleaving mRNA or through translation repression [3]. It is estimated that miRNAs can control the expression of at least 30% of all proteins in humans [4, 5], thus regulating various cellular processes. miRNAs are transcribed from either coding or non-coding genes and undergo different transcription regulation. Approximately half of the miRNAs originate from noncoding loci, while the remaining are transcribed from intragenic loci. Mature miRNA is generated through two-step cleavage process, first nuclear and subsequent cytoplasmic cleavage events [6]. miRNAs are initially transcribed from the genome by RNA polymerase II (RNAP II) into primary transcripts (pri-miRNA) and processed in the nucleus to hairpin structures of about ~70 nucleotide precursors miRNA (pre-miRNAs) by the ribonuclease (RNAase) III family enzyme Drosha. These pre-miRNAs subsequently are exported to the cytoplasm by exportin-5 [7] or exportin-1 [8] where the loop sequence is removed from the hairpin by Dicer to produce an RNA duplex analogous [9]. One strand is discarded, leaving only the ~22 nucleotide mature miRNA species. Many miRNAs are aberrantly expressed in several pathological conditions, including cancer, leading to the identification of “miRNA signatures” characteristic of certain tumors. Tumor-specific miRNA expression profiles are also functionally relevant because many miRNAs act as tumor suppressors or as oncogenes.

miR-145 may function in tumor suppression, since its expression is reduced in most human cancer cells and particularly so in aging colon and prostate cancers [10, 11]. The enforced expression of miR-145 in human colon or gastric cancer cells significantly inhibits their growth. The miR-145 in turn targets pluripotency factors such as OCT4, SOX2, and KLF4, and contributes in the processes of stem cell growth and dedifferentiation [12]. These findings indicate an anti-oncogenic role for miR-145 especially in gastrointestinal cancers. In contrast, miR-21 is over-expressed in most epithelial cancers including CRC. Knockdown of miR-21 expression in cancer cells impairs growth [13], induces apoptosis and reduces migration and invasion of cancer cells [14]. Forced expression of miR-21 leads to increased β-catenin activity, augmentation of c-Myc and Cyclin-D expression, increase in CSCs, and is accompanied by increased colonospheres forming ability in vitro and tumor formation in SCID mice [15]. The target genes of miR-21 include tumor suppressors such as PTEN, PDCD4 and TGFβR2 [15, 16]. Therefore, miR-21 is believed to play a pivotal role in the progression of many malignancies including CRC and has been called an “oncomiR”.

LncRNAs were considered as non-functional junk initially. But now, they are thought to carry out important regulatory functions, adding yet another layer of complexity to our understanding of genomic regulation [17]. LncRNAs are transcribed by RNA polymerase II, and some lncRNAs are further regulated via splicing, processing at the 5′ and 3′ ends, and exported to cytoplasm [18].

Colon cancer-associated transcript-2 (CCAT2) is a lncRNA. A significant proportion of CCAT2 is mainly located in the nucleus and can be tissue- and cell-type specific. It is a 1752 base RNA transcribed from the 8q24 region of the human genome containing the SNP (single- nucleotide polymorphism) rs6983267 [19]. The rs6983267 has been consistently associated with an increased risk of CRC [20–22] and the other cancer types, including prostate, ovarian, and breast cancer [19, 23]. Although the molecular and cellular mechanisms of increased cancer risk from this SNP variant remain largely unknown, the genomic region spanning rs6983267 was found to contain DNA enhancer elements such as those that bind to TCF7L2 (transcription factor 7-like 2), a transcription factor that, together with β-catenin, plays a central role in regulating CSC [19]. CCAT2 has also been reported to be over-expressed in multiple types of cancer including CRC, breast [24, 25], lung [26], esophageal squamous cell carcinoma [27], gastric cancers [28], and to promote tumor growth and metastasis [19, 26] while causing a reduced sensitivity to chemotherapy [25], a property related to cancer stem cells (CSCs) [29, 30].

We have investigated and confirmed that age-related increases in adenomatous polyps are associated with increases in mucosal CSCs [31] accompanied by a concomitant rise in miR-21 and reduction in miR-145 [32]. Similar results were also observed in animal model following administration of colonic carcinogen [32]. Further, we have found that there is enrichment of CSCs and dysregulation of microRNAs in chemo-resistant (CR) colon cancer cells [15, 33]. Moreover, we have recently reported that cooperation of miR-145 and miR-21 regulates colon CSCs proliferation and differentiation [34]. Here, we report that CCAT2 selectively blocks miR-145 processing, resulting in decreased mature miR-145 expression and regulation of colon CSC proliferation and differentiation.

## Results

### Regulating the expression of CCAT2 in colon cancer cells

**Fig. 1:**
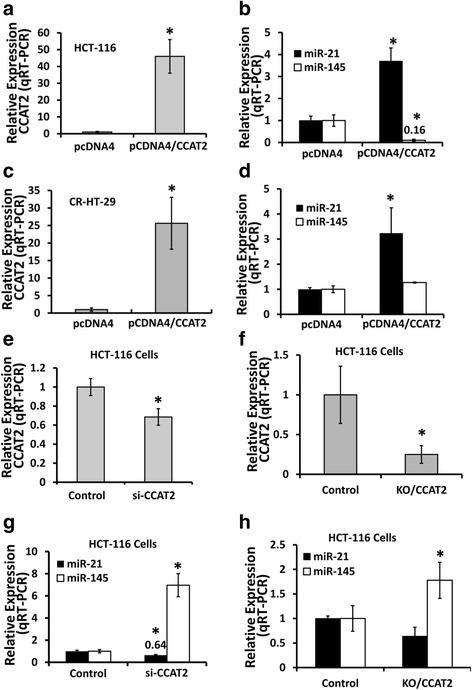
CCAT2 regulates the expression of miR-21 and miR-145 in colon cancer HCT-116 and CR-HT-29 cells. Quantitative real-time RT-PCR (qRT-PCR) showing that overexpression of CCAT2 by stable transfection of pcDNA4/CCAT2 plasmid (**a** and **c**) downregulates miR-145 and upregulates miR-21 in HCT-116 and CR-HT-29 cells (**b** and **d**). Downregulation of CCAT2 through transfection of si-CCAT2 (**e**) or by CRISPR/Cas9 (**f**) increased the expression of miR-145 and negatively regulates miR-21 (**g** and **h**). All the data represent means ± SEM, *P < 0.001, compared to the control

To determine the putative functional properties of CCAT2 in the development of colorectal tumor and its relation to miRNAs expression, pCMV/CCAT2 plasmid was stably transfected in HCT-116 and CR-HT-29 cells. As determined by qRT-PCR (real time PCR) analysis, the expression of CCAT2 was found to be ~25–50-fold higher in the CCAT2 positive cells, compared to those with the empty vector (Fig. 1a and c). We observed overexpression of CCAT2 to cause downregulation of miR-145 and to stimulate miR-21 (Fig. 1b and d).

**Fig. 2:**
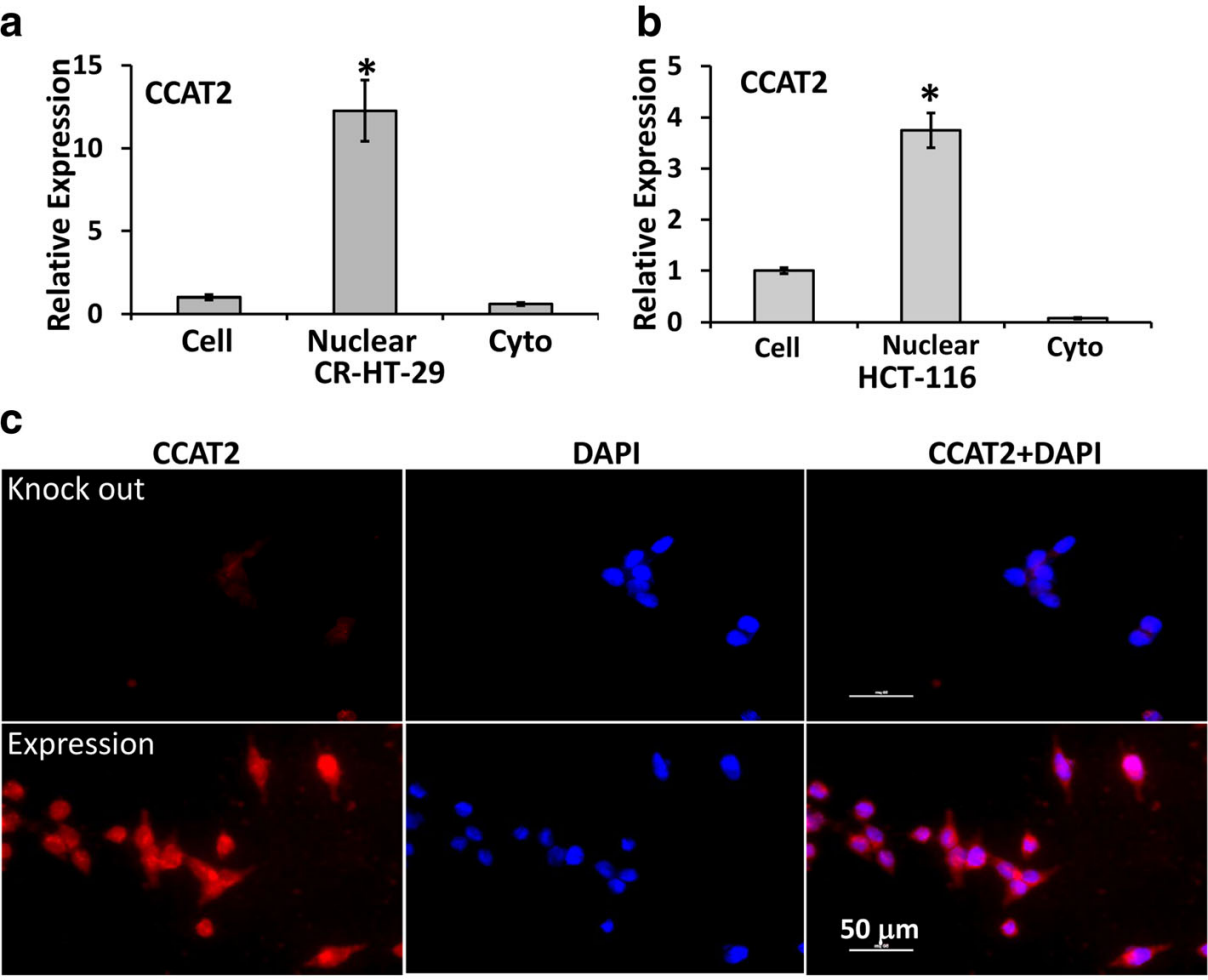
Localization of CCAT2 in CR-HT-29 or HCT-116 cells by qRT-PCR and FISH. qRT-PCR was performed with RNA isolated from nuclear and cytoplasmic fraction of stably over-expressing CCAT2 cells to determine its expression in CR-HT-29 and HCT-116 cells (**a** and **b**). All the data represent means ± SEM, *P < 0.001, compared to the control. (**c**) The representative photograph showing fluorescence in situ hybridization in CCAT2 knockout or overexpressing HCT-116 cells, red represents biotin-labeled probe against CCAT2 RNA and blue represents DAPI staining of the nucleus

The next set of experiments was carried out to determine whether knockdown or knockout of CCAT2 increased the expression of miR-145. To conduct the experiments, we utilized siCCAT2, which induced a knockdown of ~30% of CCAT2 compared with corresponding vector (NT-siRNA) controls. CCAT2 knockdown using CRISPR-CAS9, showed ∼80% reduction in CCAT2 levels compared with the corresponding controls (Fig. 1e and f). As expected, CCAT2 knockdown increased the expression of miR-145 and negatively regulated miR-21 in HCT-116 cells (Fig. 2g and h).

### CCAT2 over-expression decreases mature miR-145 expression and increases pre-miR-145

Next, we moved on to explore the mechanism of miR-145 regulation by CCAT2. Considering miRNA biogenesis process, we first determined the location of CCAT2 in colon cancer cells. RT-PCR and in situ hybridization was employed for this set of experiments. First, the nuclear and cytoplasmic fractions from stable clones of CCAT2 over-expressing colon cancer cells were isolated and the RNA was extracted from these fractions. The qRT-PCR results show that the expression of CCAT2 was >10 fold higher in the nucleus and only 0.6 fold in cytoplasmic fractions compared with corresponding chemo-resistant CR-HT-29 cells (Fig. 2a). In HCT-116 cells, CCAT2 was 3.5 time higher in nucleus (Fig. 2b). To visualize *CCAT2* and its location in the cells, we performed fluorescence in situ hybridization (FISH) by using a biotin-labeled nucleic acid probe against *CCAT2* RNA. The fluorescence was detected in both the nucleus and the cytoplasm, with a more intense fluorescence in the nucleus, indicating an obvious enrichment of *CCAT2* in the nuclear compartment (Fig. 2c).

**Fig. 3:**
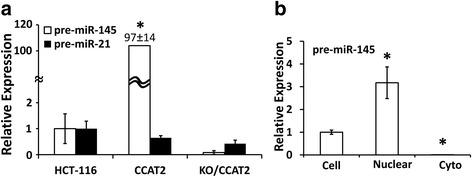
Localization of pre-miR-145 in colon cancer HCT-116 cells by qRT-PCR. qRT-PCR showing the expression of pre-miR-145 and pre-miR-21 in stable clones over-expressing or knockout CCAT2 HCT-116 cells (**a**), and the relative pre-miR-145 expression in the whole cell and in nuclear and cytoplasmic fraction in HCT-116 cells (**b**). All the data represent means ± SEM, *P < 0.001, compared to the control

In the next set of experiments we tested whether the pre-miR-145 level was associated with expression and location of CCAT2 in the modulated colon cancer cells. We observed that the expression of pre-miR-145 was 97-fold higher in stable clones expressing CCAT2, and 90% lower in CCAT2 knockout HCT-116 cells compared with corresponding HCT-116 cells. However, pre-miR-21 was decreased in both CCAT2 over-expressing and knockout cells (Fig. 3a). pre-miR-145 was enriched in the nuclear fraction of cells (Fig. 3b). The expression of CCAT2 positively correlated with expression of pre-miR-145 but negatively with mature miR-145 that indicating that CCAT2 regulates miR-145 maturation process.

### CCAT2 regulates miR-145 maturation process in vitro

**Fig. 4:**
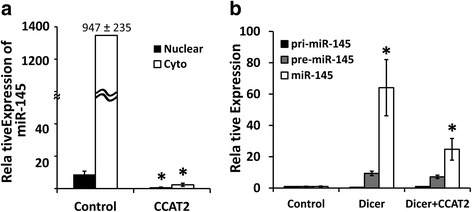
CCAT2 inhibits maturation process of miR-145. The pri-miR-145 containing pre-miR-145 and up and down-stream flanking sequence was synthesized, subsequently incubated with nuclear or cytosolic fraction in the presence or absence of CCAT2. (**a**) qRT-PCR showing the relative concentration of mature miR-145 following incubation with nuclear or cytoplasmic fractions. (**b**) The levels of pri-, pre- and mature miR-145 in the control or after digestion with recombinant human Dicer. All the data represent means ± SEM, *P < 0.001, compared to the control

**Fig. 5:**
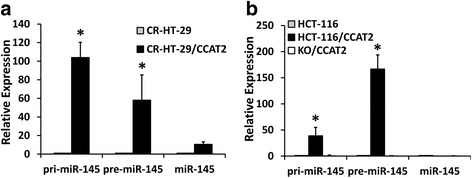
CCAT2 blocks cleavage of pri- and pre-miR-145 in vitro. The qRT-PCR results showing pri-, pre- and mature miR-145 levels following recombinant Dicer reaction containing the total RNAs isolated from either CCAT2 overexpressing or CCAT2-KO (**a**) CR-HT-29 or (**b**) HCT-116 cells. All the data represent means ± SEM, *P < 0.001, compared to the control

**Fig. 6:**
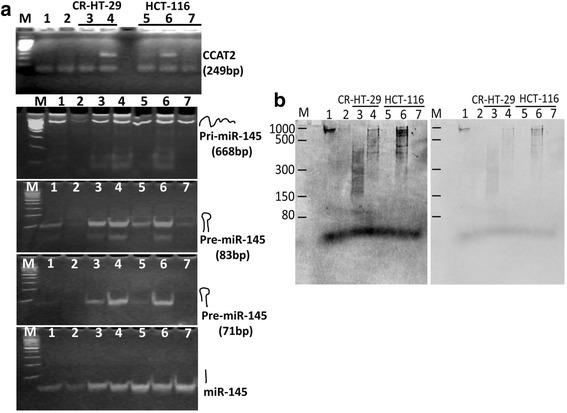
CCAT2 regulates miR-145 expression by directly suppressing its processing. Digoxigenin labeled 200 ng of pri-miR-145 (668 base) containing 1 μg of total RNAs from the CCAT2 overexpressing or knockout (KO) colon cancer cells was incubated at 25 °C for 5 min; 1unit of recombinant Dicer was added and incubated at 37 °C for 60 min. The samples were divided into two parts: (**a**) performed RT-PCR to detect CCAT2, pri-, pre- and mature miR-145. Agarose (3%) chromatography demonstrates changes in PCR products. (**b**) carried out electrophoresis on 8% polyacrylamide-8M urea gel, subsequently transferred onto membrane and the Dig-labeled RNAs detected with anti-digoxigenin antibody. M: 100 bp DNA (A) or RNA ladder (B), Lane-1: pri-miR-145, Lane-2: pri-miR-145 + Dicer, Lane-3: pri-miR-145 + RNA of CR-HT29 + Dicer, Lane-4: pri-miR-145 + RNA of CR-HT29/CCAT2 + Dicer, Lane-5: pri-miR-145 + RNA of HCT-116 + Dicer, Lane-6: pri-miR-145 + RNA of HCT-116/CCAT2 + Dicer, Lane-7: pri-miR-145 + RNA of HCT-116/KO-CCAT2 + Dicer

Based on above results, we hypothesized that CCAT2 downregulates miR-145 expression by selectively suppressing its maturation process in colon cancer cells. To test this hypothesis, the pri-miR-145 containing pre-miR-145 and up and down-stream flanking sequence (total 668 nucleotide) was synthesized in vitro by T7 RNA polymerase and labeled by incorporation of digoxigenin-UTP. First, the synthesized pri-miR-145 was digested by the nuclear or cytoplasmic fractionations of HCT-116 cells separately. The enzyme activities in these fractions were maintained to provide conditions similar to those in the cellular environment. The mature miR-145 was highly increased in the reaction containing cytoplasmic fraction (Fig. 4a), which agrees with an earlier observation [35]. Alternately, the synthesized pri-miR-145 was cleaved by recombinant human Dicer enzyme with or without CCAT2. The results of qRT-PCR show that in the presence of CCAT2 the expression of miR-145 was decreased by more the 50% (Fig. 4a and b). Finally, the dig-labeled pri-miR-145 was mixed with the RNA which was isolated from over-expression or knock out CCAT2 colon cancer CR-HT-29 and HCT-116 cells or corresponding controls, and digested by recombinant human Dicer enzyme. The relative levels of pri-, pre- and mature miR-145 within individual reaction are listed in Table 1. qRT-PCR shows that the pri-miR-145 was ~35–100 fold higher in reaction containing RNA from CCAT2 over-expressing cells compared with the corresponding controls (Fig. 5a and b). Pre-miR-145 was determined by the two sets of primers and the resulting PCR products were 83 bp and 71 bp, separately. The results of agarose gel clearly show that the level of pre-miR-145 correlated with CCAT2 in the reactions (Fig. 6a).

**Table 1:** qRT-PCR showing the relative levels of pri-, pre- and mature miR-145. The reaction mixture contained synthesized pri-miR-145 and total RNAs isolated from CR-HT-29 or HCT-116 CCAT2 overexpressing or CCAT2-KO cells, and corresponding controls digested with Dicer

	Pri-	Pre-	Mature
HCT-116 Cells:			
Control	1	0.14	613.11
CCAT2	1	0.61	0.18
KO/CCAT2	1	0.08	215.27
CR-HT-29 Cells:			
Control	1	0.29	0.65
CCAT2	1	0.16	0.07

To avoid the effect of endogenous production of pri-, pre- and miR-145, the RNAs digested by Dicer were also separated on denaturing polyacrylamide gel, transferred to the PVDF membrane and detected with anti-digoxigenin antibody. The image shows that Dicer cleaves more than ~80–90% of Dig-labed pri-miR-145 (Fig. 6b lane 1 and 2), and RNA which comes from CCAT2 overexpressing clones (Fig. 6b lanes 4 and 6) suppressed the digestion reaction compared with the corresponding controls (lanes 3 and 5) or CCAT2 knockout HCT-116 cells (lane 7).

## Discussion

Recent studies have implicated that long non-coding RNAs (lncRNAs), a new class of regulatory RNA, play a key role in regulating development and growth of a tumor. Despite recent insights into how lncRNAs function in such diverse cellular processes as regulation of gene expression and assembly of cellular structures, the key questions regarding lncRNA mechanisms remain to be answered [2, 36]. lncRNA CCAT2 is over-expressed in colorectal cancer and promotes tumor growth, metastasis and reduces sensitivity to chemotherapy that is associated with colon CSC and regulated by cooperation of miR-145 and miR-21 [34, 37]. In order to reveal whether and how CCAT2 regulates the expression of miRNAs −145 and −21 in colon CSC, we stably over expressed CCAT2 in HCT-116 and CR-HT-29 cells. We noted a down regulation of miR-145. On the other hand, knockdown of CCAT2 by siRNA or by CRISPR/Cas9 increased the expression of miR-145. Taken together, our results suggest that CCAT2 negatively controls the expression of miR-145. However, the reason for disproportionate change in miR145 levels between the transient siRNA transfection and stable KO using CRISPR/Cas9 is not clear. It is possible that some other regulatory processes get activated in stable transfection.

**Fig. 7:**
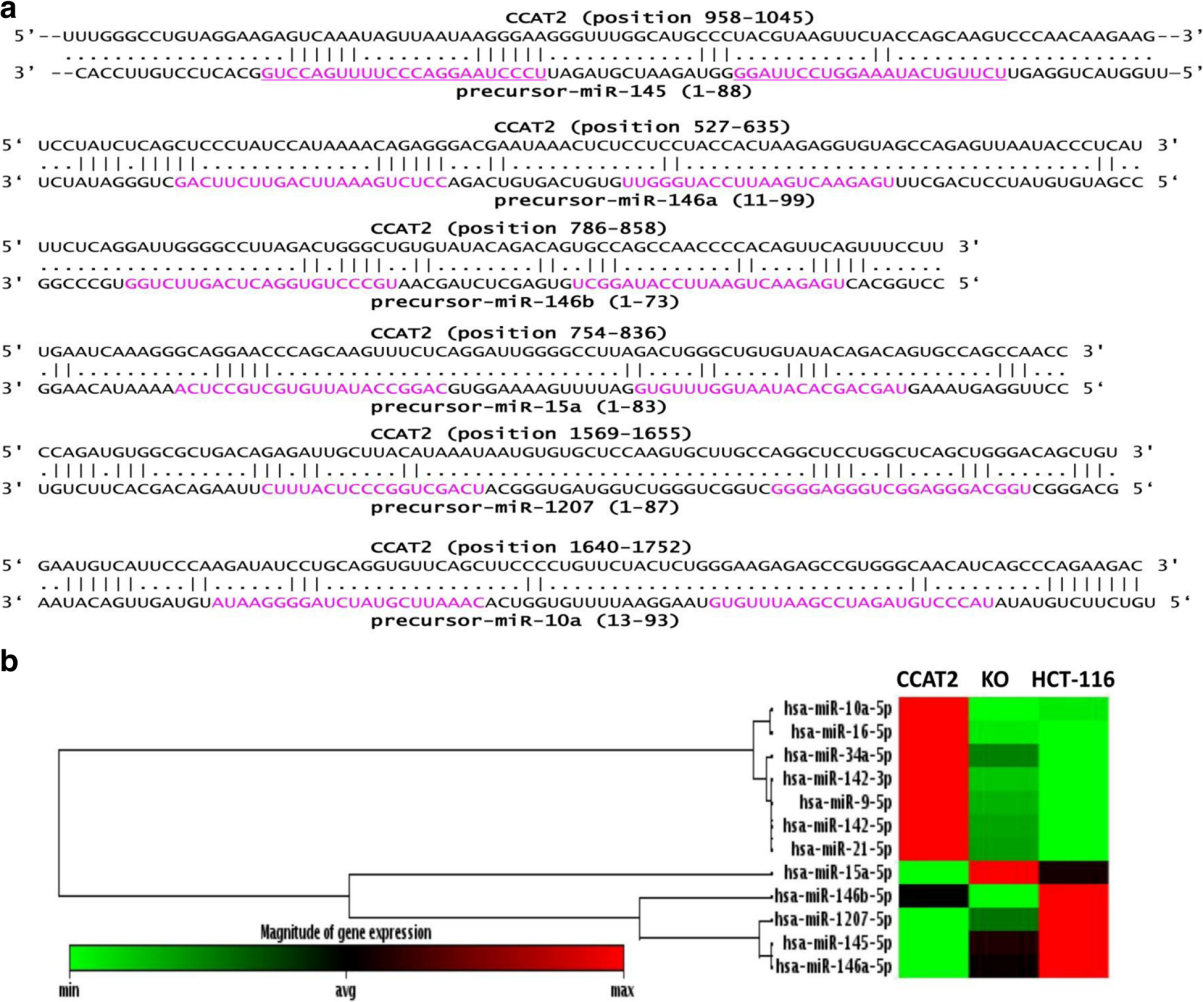
The putative CCAT2 binding to precursor miRNAs. (**a**) A schematic representation of the putative binding sites in precursor miR-145/146a/146b/15a/1207/10a that indicates CCAT2 might regulate the processing of miRNAs. The pink letters indicate mature sequence of the miRNAs. (**b**) RT-PCR based miRNA expression profiles in overexpressing, knockout CCAT2 and control (wild type) HCT-116 cells

To expose the mechanism of CCAT2 regulating miR-145, we have analyzed the location of CCAT2 in vivo and in vitro. Considering the biogenesis of miR-145, we used bioinformatics tool LncTar (38) to predict CCAT2 interaction with pre-miR-145 (Fig. 7a)*.* CCAT2 is enriched in the nucleus and correlates with expression of pre-miR-145 but not pre-miR-21, which implies that CCAT2 may selectively produce or block export of pre-miR-145 to cytoplasm. Moreover, we revealed that CCAT2 blocks cleavage of DIG-Labeled pri-miR-145 (pre-miR-145 with 250–300 nts up and down-stream flanking sequence) in cell extract or by recombinant Dicer in vitro (Figs. 4, 5 and 6). Together, the results from current investigation show that CCAT2 selectively blocks miR-145 biogenesis process, resulting in decreased mature miR-145 expression. These data demonstrate that CCAT2 interacts with nascent miR-145 and inhibits its maturation process. Various other miRNAs such as *pre-miR146ab,pre-miR-15a, pre-miR-1207 and pre-miR-10a* may also be affected by CCAT2 via similar mechanisms as predicted, and by our miRNA PCR array data (Fig. 7).

While it has recently become apparent that a cross regulation exists between miRNAs and lncRNAs, which include miRNA triggered lncRNA decay, lncRNAs as miRNA sponges/decoys, lncRNAs competing with miRNAs for interaction with mRNAs and lncRNAs generating miRNAs. We speculated that as transcription is the first step of gene expression in nucleus, the RNA may be further processed either in the nucleus or exit to the cytoplasm through the nuclear pore complex. Therefore, we hypothesized that the nascent RNAs interaction should be the first layer of lncRNA regulating gene expression including miRNA. Our current data demonstrating that CCAT2 blocks miR-145 maturation process resulting in reduced mature miR-145, has supported our hypothesis and exposed a novel mechanism for lncRNA and miRNA crosstalk.

To more clearly understand the mechanism of CCAT2 regulated expression of miRNAs, we used bioinformatics tool LncTar [38] and predicted CCAT2 to regulate expression of some of the miRNAs related to growth and differentiation of CSC, such as miR-146a [19], miR-146b, miR-10a, miR-15a, miR-1207, miR-16. PCR based miRNA array showed that CCAT2 may interact with miR-146a/146b/15a/1207 but not with miR-16 and miR142 (Fig. 7).

LncRNA CCAT2 can influence diverse cellular processes by directly and/or indirectly regulating the gene expression. Ling et al. reported that *MYC* is up-regulated by *CCAT2* through TCF7L2-mediated transcriptional regulation and via MYC regulated miRNAs such as miR-17-5p and miR-20a [19]. Recently, their studies have shown that CCAT2 interaction with the Cleavage Factor I complex fine-tunes the alternative splicing of Glutaminase (*GLS*) by selecting the poly(A) site in intron 14 of the precursor mRNA [39].

During miRNA biogenesis, Exportin-5 [7] and Exportin-1 [8] transport miRNA precursors from the nucleus to the cytoplasm. The exportin-5 was identified as a nuclear export factor of pre-miRNA but recent study using exportin-5 knockout cells confirms the involvement of exportin-5 in the miRNA biogenesis pathway but it is not an essential factor for miRNA biogenesis, at least in HCT116 cells [9]. Exportin-1, the cell-cycle-regulated gene encodes a protein, instead of exprotin-5 that exports Drosha-, exportin-5- and Dicer- independent pre-miRNAs from the nucleus to the cytoplasm [8, 9]. Whether CCAT2 directly affects the expression and function of either exportin-5 or exportin-1 is unclear. However, it is enriched in the nucleus and correlates with the expression of pre-miR-145 but not pre-miR-21(Fig. 3a) which indicates that CCAT2 selectively inhibits some pre-miRNA export to cytoplasm.

In the current investigation, we have focused our attention on CCAT2 and miR-145 interaction. The rationale for analyzing CCAT2 and miR-145 cooperation is because CCAT2 promotes tumor growth and metastasis [19, 26], causing a reduced sensitivity to chemotherapy [25] which is the property related to cancer stem cells (CSCs) [29, 30]. In addition, miR-145 and miR-21 regulate stem cell growth and dedifferentiation via their targets, OCT4, SOX2, NANOG and TGFβR2 [12, 15]. Thus, we examined miR-21 and miR-145 levels in CCAT2 overexpressing colon cancer cells as well as in knock down/knockout cells. Interesting, we also observed that expression of CCAT2 can regulate CSC function, which was evident by the observation that overexpression of CCAT2 increases the expression of CD44 and SOX2, reduces the expression of CK-20 and causes cells to detach from the dish resulting in increased number of cells in suspension (Additional file 1: Figure S1); the colonosphere forming ability was significantly augmented. The opposite results were found in the CCAT2 knockout colon cancer cells. These phenomena agree with previous reports that CCAT2 promotes tumor metastasis.

## Conclusions

In conclusion, our study demonstrates that CCAT2 selectively blocks miR-145 maturation process by inhibiting pre-miR-145 export to cytoplasm and blocks cleavage of pre-miR-145 in cell extract or by Dicer in vitro. The results imply lncRNA CCAT2 as a negative regulator of miRNA-145 biogenesis, and expose a novel mechanism of lncRNA-miRNA crosstalk.

## Methods

### Cell lines and cell cultures

Human colon cancer HCT-116 and HT-29 cells were obtained from the American Type Culture Collection (ATCC, Rockville, MD). Cells were maintained in Dulbecco’s modified Eagle medium (DMEM; 4.5 g/L d-glucose) supplemented with 10% FBS (Invitrogen, Grand Island, NY) and 1% antibiotic/antimycotic in tissue culture flasks. 5-Fluorouracil and Oxaliplatin (Fu-Ox) resistant [chemo-resistant (CR)] colon cancer HCT116 and HT29 cells were generated as described earlier [15, 33] in our laboratory and were maintained in normal culture medium containing 2× FuOx (50 μM 5-Fu + 1.25 μM Ox) in tissue culture flasks in a humidified incubator at 37 °C in an atmosphere of 95% air and 5% carbon dioxide. The medium was changed two times a week, and cells were passaged using 0.05% trypsin/EDTA (Invitrogen, Grand Island, NY).

### Generation of CCAT2 over-expressing HCT-116, CR-HT-29 cells

pCDNA-CCAT2 plasmid or empty vector DNA alone was transfected into HCT-116 and CR-HT-29 cells by Lipofectamine™ 2000 reagent according to manufacturer’s instructions (Invitrogen Corp, CA). Several independent sublines (colonies) were generated over 8–10 wk of the selection period in the presence of 0.4 mg/ml G418 (Neomycin). Colonies were collected and grown as individual cell lines in the presence of 0.4 mg/ml G418. The cells were subjected to RT-PCR analysis to evaluate CCAT2 expression.

### Knock out CCAT2 in HCT-116 cells by CRISPR-CAS9

The plasmid vector expressing Cas9 enzyme driven by the CMV promoter and an enhanced GFP-selectable marker was obtained from OriGene (plasmid GE100018, Rockville, MD). CRISPR guide RNA specifically targeting CCAT2 sequence (210–219 Forward) and (1288–1307 Reverse) was cloned into the vector, (forward gRNA GAGCTAAGAGGAAACCACCT and complement strand AGGTGGTTTCCTCTTAGCTC; reverse gRNA CTCCTATTCATACCATATTA and complement strand TAATATGGTATGAATAGGAG) and verified by DNA sequencing. HCT-116 cells were transfected using Lipofectamine 3000 transfection reagent (Invitrogen Corp, CA), and positive cells were sorted for enhanced GFP by flow cytometry and directly seeded into the 96 well plates as single cell per well. Two week after transfection, single clones were isolated and expression of CCAT2 was detected by RT-PCR.

### Isolation of nuclear and cytoplasmic fractions

Nuclear–cytoplasmic fractionation was conducted using the NE-PER Nuclear and Cytoplasmic Extraction Reagents kit (Thermo Fisher Scientific) according to the manufacturer’s protocol. Briefly, the cells were washed with PBS and suspended in 200 μl of cytoplasmic extraction reagent I, 11 μl of cytoplasmic extraction reagent II was added subsequently, vortexed for 5 s, incubated on ice for 1 min and centrifuged for 5 min at 16000 *g*. The supernatant fraction (cytoplasmic extract) was transferred to a pre-chilled tube. The pellet fraction contains crude nuclei.

### In situ hybridization of CCAT2

The knock out or overexpressing CCAT2 HCT-116 cells were grown in a 4 chamber slide for 48 h. After washing with PBS, the cells were fixed with 3.7% paraformaldehyde, permeabilized with 70% ethanol, and rehydrated in 2×SSC with 50% formamide for 5 min at room temperature. Cells were hybridized overnight at 42 °C with biotin-labeled CCAT2 probe mixture containing 10% dextran sulfate, 5X Denhardt’s reagent, 2× SSC, 50% formamide and 100 μg/ml denatured fragmented salmon sperm DNA. The non-specific probe was removed by 0.5× SSC containing 50% formamide at 37 °C. The anti-biotin monoclonal antibody and Alexa Fluor® 647–conjugated secondary antibody were used for detecting biotin-labeled CCAT2. Cells were washed with PBS and then placed on cover slips with prolong gold antifade reagent containing DAPI (Cell Signaling Technology, Boston, MA, USA). Stained cells were observed under an Olympus microscope supporting a Hamamatsu 1394 ORCA-ERA video camera and the images were stored using Slidebook Digital Microscopy Software (Intelligent Imaging Innovations).

### DIG-labeling of pri-miR-145

pCMV-miR-145 plasmid carrying pre-microRNA-145 and 250–300 nts up and down-stream flanking sequence (Origene, Nockville, MD) was linearized with the restriction enzyme (Not I and Xho I) to make a template for in vitro transcription. The pri-miR-145 was synthesized and labeled by incorporation of digoxigenin-UTP (Roche Molecular Biochemicals, Indianapolis, IN) using a Maxiscript T7 in vitro transcription kit (Invitrogen).

### In vitro processing of pri-miRNAs

In vitro processing of pri-miRNAs was performed using recombinant human Dicer enzyme kit (Genlatis, San Diego, CA) [40]. Briefly, 10 μL of processing reaction contained 4ul dicer reaction buffer, 2 μL of recombinant dicer enzyme, 2.5 mM MgCl_2_, 1 mM ATP, and 0.2 μg of Digxigenin labeled pri-miR-145. The reaction mixture was incubated at 37 °C for 90 min. RNA was extracted from the reaction mixture by phenol extraction and was assessed by quantitative RT-PCR for determination of mature miR-145, pre-miR-145 and pri-miR-145. The RNA was also analyzed on 8% denaturing polyacrylamide-8M urea gel, transferred to PVDF membranes and detected with anti-digoxigenin antibody.

### Isolation of RNA and quantitative polymerase chain reaction analysis

Total RNA was extracted from cells, nuclear and cytoplasmic fractions using RNA-STAT solution (Tel Test, Friendswood, TX) according to the manufacturer’s instructions. The total RNA was treated with DNase I and purified with phenol-chloroform. RNA concentration was measured spectrophotometrically at an optical density of 260 nm.

Quantitative reverse transcription-polymerase chain reaction (qRT-PCR) was performed using the GeneAmp RNA PCR Kit (Applied Biosystems, Foster City, CA). 5 μl of cDNA products were amplified with SYBR Green Quantitative PCR Master Mix (Applied Biosystems). PCR primers were used as follows: pri-miR-145, forward: 5′-ccaggctaggaactgaatgg-3′, reverse: 5′-caagaaacgcatgcctgat-3′; pre-miR-145-1, forward: 5′-cttgtcctcacggtccagtt-3′ and reverse: 5′-ccatgacctcaagaacagtatttct-3′, pre-miR-145-2 forward: 5′-gtccagttttcccaggaa-3′ and reverse: 5′- ccatgacctcaagaacagtatttct −3′; CCAT2, forward: 5′-tgctccaggcaataactgtg-3′ and reverse: 5′-gaaagtgggctcattccttg-3′; β-actin forward: 5′-cccagcacaatgaagatcaa-3′ and reverse 5′-acatctgctggaaggtggac-3′. Reactions were carried out in Applied Biosystems 7500 Real-Time PCR System. The running conditions for PCR were as follows: for activating the DNA polymerase, hot start was performed for 10 min at 95 °C, and then cycling at 95 °C for 15 s and 60 °C for 1 min for a total of 40 cycles.

### Quantitation of miRNA-21 and miR-145

TaqMan microRNA assays were used to quantitate miR-21 and miR-145 in different colon cancer cells according to the manufacturer’s instruction (Applied Biosystems, Foster City, CA). Briefly, cDNA synthesis was carried out with the TaqMan MicroRNA reverse transcription kit (Applied Biosystems). The miRNA reverse transcription-PCR (RT-PCR) primers for miR-21, miR-145 and endogenous control RNU6B were purchased from Applied Biosystems. Real-time quantitative RT-PCR (qRT-PCR) analysis was carried out using Applied Biosystems 7500 Real-time PCR System. The PCR mix containing TaqMan 2× Universal PCR Master Mix were processed as follows: 95 °C for 10 min and then 95 °C for 15 s, 60 °C for 60 s for up to 40 cycles. Signal was collected at the endpoint of every cycle. The gene expression Δ*C*_T_ values of miRNAs from each sample were calculated by normalizing with internal control RNU6B and relative quantitation values were plotted.

### Formation of colonospheres and extreme limiting dilution analysis

The ability of miR-145-overexpressing and parental HCT-116 cells to form spheres in suspension was evaluated as described previously [41].

### Statistical analysis

Unless otherwise stated, data are expressed as mean ± SEM. Wherever applicable, the results were analyzed using analysis of variance followed by Fisher protected least significant differences or Scheffé test. *p* < 0.05 was designated as the level of significance.

## Additional file

Additional file 1: Figure S1. Additional Figure and legend. (DOCX 560 kb)

## Abbreviations

CCAT2: Colon cancer-associated transcript-2
CRC: colorectal cancer
CSC: stem or stem-like cells
FISH: fluorescence in situ hybridization
lncRNA: long noncoding RNA
pre-miRNA: precursors miRNA
pri-miRNA: primary miRNA
qRT-PCR: quantitative reverse transcription-polymerase chain reaction
SNP: single-nucleotide polymorphism
