# Supplementary material for: A novel mechanism of lncRNA and miRNA interaction: CCAT2 regulates miR-145 expression by suppressing its maturation process in colon cancer cells
Source: Mol Cancer. 2017 Sep 30;16:155. doi: 10.1186/s12943-017-0725-5 (PMC5622467; doi:10.1186/s12943-017-0725-5)
Supplement: Additional file 1: Figure S1. — Additional Figure and legend. (DOCX 560 kb) [file 12943_2017_725_MOESM1_ESM.docx]

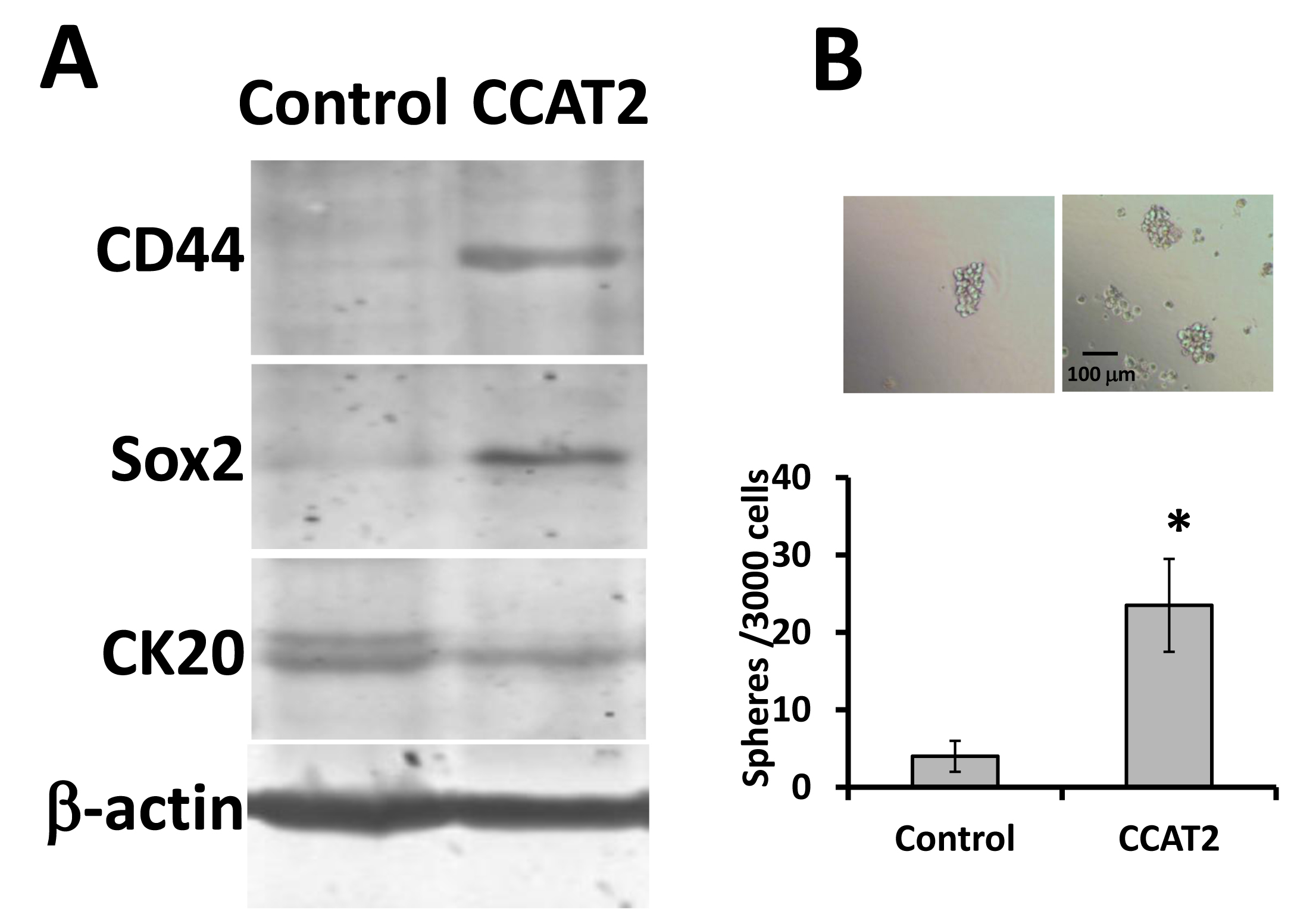


Fig 1. Over-expression of CCAT2 in HCT-116 cell through stable transfection of pcDNA/CCAT2 increases the expression of CD44 and SOX2, the direct target of miR-145, and reduces the expression of CK20 **(A)**.

**(B)** Representative photographs showing colonospheres formed by the flooding cells from CCAT2 expressing clones, derived from CR-HT-29 cells, stably transfected with pcDNA/CCAT2 plasmid or the corresponding vector (left top panel). The histogram showing the number of colonspheres formed by detached CCAT2 over-expressing CR-HT-29 cells from condition medium, compared to the corresponding vector-transfected control cells ( left bottom panel, *P < 0.001).
